# Multimass Analysis of Adeno-Associated Virus Vectors by Orbitrap-Based Charge Detection Mass Spectrometry

**DOI:** 10.1021/acs.analchem.4c05229

**Published:** 2024-10-10

**Authors:** Ryoji Nakatsuka, Yuki Yamaguchi, Kiichi Hirohata, Saki Shimojo, Makoto Murakami, Mark Allen Vergara Rocafort, Yasuo Tsunaka, Mitsuko Fukuhara, Tetsuo Torisu, Susumu Uchiyama

**Affiliations:** † Department of Biotechnology, Graduate School of Engineering, Osaka University, 2-1 Yamadaoka, Suita, Osaka 565-0871, Japan; ‡ Technology Research Laboratory, Shimadzu Corporation, 1 Nishinokyo-Kuwabaracho, Nakagyo-ku, Kyoto 604-8511, Japan; § Osaka University Shimadzu Analytical Innovation Research Laboratories, Osaka University, 2-1 Yamadaoka, Suita, Osaka 565-0871, Japan; ∥ U-Medico Inc., 2-1 Yamadaoka, Suita, Osaka 565-0871, Japan

## Abstract

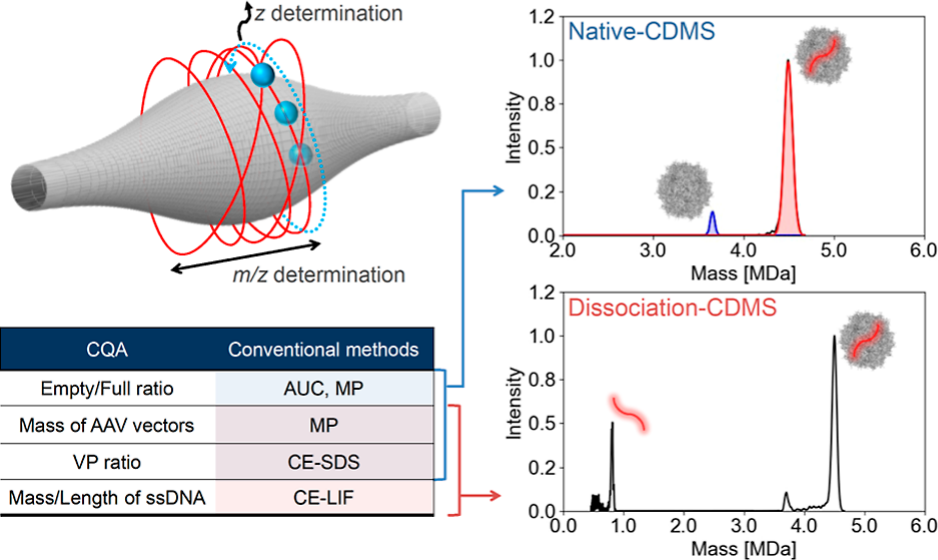

Adeno-associated virus (AAV) vectors have attracted significant attention as the main platform for gene therapy. To ensure the safety and efficacy of AAV vectors when used as gene therapy drugs, it is essential to assess their critical quality attributes (CQAs). These CQAs include the genome packaging status, the size of the genome encapsidated within the AAV capsid, and the stoichiometry of viral proteins (VPs) that constitute the AAV capsids. Analytical methods have been established for evaluating CQAs, such as analytical ultracentrifugation, capillary gel electrophoresis with laser-induced fluorescence detection, and capillary gel electrophoresis using sodium dodecyl sulfate with UV detection. Here, we present a multimass analysis of AAV vectors using orbitrap-based charge detection mass spectrometry (CDMS), a single-ion mass spectrometry. Orbitrap-based CDMS facilitates the quantitative evaluation of the genome packaging status based on the mass distribution of empty and full particles. Additionally, we established a novel method to analyze the encapsidated genome directly without pretreatment, such as protein digestion or heat treatment, and to estimate the stoichiometric variation of VP for the capsid based on the mass distribution constituted by the single peak corresponding to AAV particles. Orbitrap-based CDMS is a distinctive method that allows multiple mass characterizations of AAV vectors with a small sample volume of 20 μL for 10^13^ cp/mL in a short time (30 min), and it holds the potential to become a new standard method in the assessment of CQAs for AAV vectors.

## Introduction

In recent years, with the development of gene therapy drugs, adeno-associated virus (AAV) vectors have played a pivotal role in *in vivo* gene therapy.^1,2^ Full particles (FP) of AAV have a 60-mer capsid structure primarily composed of viral protein (VP) 1, VP2, and VP3 with molecular weights of approximately 81, 66, and 59 kDa, respectively.^3,4^ The capsids of wild-type AAV typically encapsidate a genome of up to 4.7 kilobases in length. The advantages of using AAV as a vector include its low pathogenicity to humans, long-term gene expression in nondividing cells, and tissue specificity per serotype.^5,6^ Meanwhile, one of the challenges in the current manufacturing of AAV vectors is that, in addition to FP, the product includes empty particles (EP) not encapsidating genome, partial particles (PP) containing DNA fragments, and overpackaged particles (OP) containing an excess of DNA beyond the full length of the genome, even after a purification procedure.^7,8^ Gao et al. suggested that the mixture of EP and FP enhanced antigen presentation by increasing the capsid dose provided by EP using a mouse model.^9^ Pei et al. also suggested that the capsid presentation from EP was dose-dependent and higher than that from FP at the early phase of administration using a mouse model.^10^ Additionally, recent studies have revealed that AAV vector products are mixtures of FP with different VP stoichiometry and that the stoichiometric variation of VP of the capsid influences the transduction efficiency.^11−13^ Therefore, for the quality control of AAV vectors, the Food and Drug Administration guidelines describe various physicochemical characteristics as the critical quality attributes (CQAs) that influence the efficacy or safety of AAV vector pharmaceuticals, including the percentage of the total amount of EP, PP, and OP relative to the total amount of EP, PP, OP, and FP, encapsidated genome length, and VP stoichiometry. Currently, various methods have been developed for analyzing the CQAs of AAV vectors.^14−16^ The capsid content distribution is commonly evaluated by analytical ultracentrifugation (AUC),^17,18^ mass photometry (MP),^19^ ion trap-based charge detection mass spectrometry (CDMS),^20,21^ and orbitrap-based CDMS.^22,23^ Orbitrap-based CDMS, a recently developed single-ion analysis method using Orbitrap QExactive-UHMR marketed as Direct Mass Technology (DMT) mode, determines the charge from the image charge^24^ of ions in the analyzer part.^25−27^ Although several studies have reported analyses of the capsid content distribution,^22,23^ important parameters for determining the percentage of EP to the total amount of EP and FP (E/F ratio), including the limit of quantification (LOQ), have not been clarified. Additionally, owing to advances in the purification process, improvement of the purity in AAV vectors has been realized, whereas recent studies reported that the incomplete removal of EP can have an adverse effect on antigen presentation.^9,10,28^ There is thus a need for accurate and quantitative evaluation of the E/F ratio in order to create AAV vector products with low concentrations of EP. However, the limit of relative quantification (LORQ) for analyzing EP in AAV vectors by using CDMS has not been elucidated. Encapsidated genome length is evaluated using capillary gel electrophoresis with laser-induced fluorescence detection (CE-LIF)^11,29^ and ion trap-based CDMS.^30^ Currently, DNA analysis by CE-LIF and CDMS requires pretreatment. This pretreatment process can potentially induce structural changes in the encapsidated genome. It is thus crucial to establish methods that can directly measure the encapsidated genome. VP stoichiometry is evaluated by capillary gel electrophoresis using sodium dodecyl sulfate (CE-SDS) with UV detection.^11,29^ As described above, to evaluate multiple CQAs for AAV vectors, it is necessary to perform various analyses for each of them. To accelerate the development of gene therapeutics, a method to evaluate multiple CQAs in a single analysis is desired.

In this study, the LORQ of the E/F ratio in orbitrap-based CDMS was determined and compared with that of AUC and MP. Additionally, we investigated the evaluation of CQAs of AAV encapsidating genomes of three different lengths using CDMS. The encapsidated genome was directly analyzed without the pretreatment process by intentionally disassembling the AAV capsids in a low-vacuum region. We also succeeded in estimating the stoichiometric variation of VP by accurately measuring the mass distribution of each component. Comparison of the genome mass obtained from CDMS measurement with the result from CE-LIF and comparison of the VP stoichiometry obtained from CDMS measurement with the result from CE-SDS were conducted. These results demonstrate that orbitrap-based CDMS can evaluate the encapsidated genome size and the VP stoichiometry in addition to the mass of AAV and the E/F ratio and can become a new standard method in the assessment of CQAs for AAV vectors.

## Experimental Section

### AAV Vector Preparation

AAV8-CMV-EGFP used for the evaluation of accuracy and LORQ for E/F ratio assessment, and AAV8-Factor IX Padura (FIXp) and AAV8-FIXp hFIX mini-intron used for the multimass analysis were produced in-house. HEK293F cells (Viral Production Cells 2.0; Thermo Fisher Scientific, Waltham, MA) were cultured in 200 mL of BalanCD HEK293 medium (FUJIFILM Irvine Scientific, Santa Ana, CA) with 6 mM l-glutamine (FUJIFILM, Tokyo, Japan) and underwent triple transfection with the gene of interest (GOI) plasmid (CMV-EGFP, FIXp, or FIXp hFIX mini-intron), helper plasmid, and pAAV-Rep&Cap (serotype 8) plasmid combined with FectoVIR-AAV (Polyplus, Illkirch, France). After transfection, cells were lysed to release AAV particles, which were then purified by affinity chromatography and cesium chloride (CsCl) density gradient ultracentrifugation (DG-UC) in an Optima XE-90 (Beckman Coulter, Inc., Brea, CA). For the multimass analysis, laboratory-grade AAV8-histone H4 (H4C1) vectors containing an ssDNA of 1487 bases were purchased from Vector Builder (Chicago, IL). For the purposes of charge calibration, AAV8-VP3-only capsids were produced with and without the GOI plasmid. In the transfection, pAAV-Rep&Cap plasmid-mutated VP1 and VP2 start codons were used with reference to a previous study.^31^ After purification, the AAV vector sample was subjected to CE-SDS, and it was confirmed that the capsid was composed of only VP3, as shown in Figure S1. These AAV8-VP3-only-EP serve as a valuable tool for calibrating CDMS measurements due to their well-defined and uniform structure.^32^

### UV Measurements

UV measurements of absorbance at 260 and 280 nm were implemented to determine the FP concentrations using Lambda850 (PerkinElmer, Waltham, MA).^33^ The molar concentration of FP capsid is given by the following formula
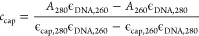
where *A*_260_ and *A*_280_ are the UV absorbances at 260 and 280 nm, respectively. At these wavelengths, the extinction coefficients for capsids and DNA are denoted as ϵ_cap,260_, ϵ_cap,280_, ϵ_DNA,260_, and ϵ_DNA,280_, respectively. The concentration of EP capsid was calculated by dividing the absorbance at 280 nm by the molar extinction coefficient. The extinction coefficients of capsid, ϵ_cap,260_ and ϵ_cap,280_, were calculated as 3.51 × 10^6^ and 6.01 × 10^6^ M^–1^ cm^–1^, respectively, from the data acquired by SV-AUC using AAV8-EP. The extinction coefficients of DNA, ϵ_DNA,260_ and ϵ_DNA,280_, were 20.0 and 11.1 g^–1^ cm^–1^, respectively.^34^

### Sample Preparation for AUC, MP, and CDMS Analyses

To evaluate the accuracy of E/F ratio determination in AAV vectors by BS-AUC, MP, and CDMS analyses, AAV vector samples at predetermined proportions (0%, 5%, 10%, 15%, 20%, 40%, 60%, 80%, and 100%) were prepared using the diluent buffer that included 1 × PBS with 0.001% poloxamer-188 and 200 mM NaCl. AAV vectors or empty capsid purified by two rounds of DG-UC were defined as FP 100% or EP 100% samples, respectively. Each sample was diluted with the buffer to achieve an absolute FP concentration of 1.0 × 10^13^ cp/mL.

### Orbitrap-Based CDMS

Prior to CDMS analysis, 20 μL of each sample was buffer-exchanged by dilution in 200 mM ammonium acetate with a Micro Biospin6 Column (Bio-Rad Laboratories, Inc.). Eight microliters of each sample was loaded into an in-house Au-coated nanocapillary tube, which was attached to the native interface mounted on the Orbitrap-Q Exactive UHMR (Thermo Fisher Scientific, Bremen, Germany). As for the instrument parameters in the ion source, spray voltage was set to 1.0–1.5 kV and capillary temperature was set to 320 °C. As for the parameters in the vacuum chamber, in-source trapping (IST) voltage was optimized within the range of −40 to −5 V and set at −15 V, and trapping gas pressure was set at 1 to 2 to achieve pressure in the range of 2.0 × 10^–8^ to 3.0 × 10^–8^ Pa using SF_6_ gas. CDMS spectrum was acquired at 50 k resolution, and ion flux was controlled by setting the manual injection time to 500 ms. Both the detector *m*/*z* optimization and the ion transfer target *m*/*z* were set at “high”. Acquisition time of CDMS was from 20 to 50 min. Acquired CDMS data were processed by STORIBoard software (Proteinaceous, Chicago, IL).^25^ GroEL (Sigma-Aldrich, St. Louis, MO) and AAV8-VP3-only-EP (in-house) were used for determining the charge calibration coefficient. After the samples had been buffer-exchanged, the *m*/*z* spectrum for each sample was acquired, as shown in Figure S2. AAV vector samples with predetermined proportions were used to evaluate the accuracy of relative quantitation and to assess the LORQ. In this study, the concentration of FP was fixed at 1.0 × 10^13^ or 4.5 × 10^13^ cp/mL, regarding EP as an impurity. CDMS data were acquired three times, and the standard deviation of relative quantitation was calculated for each proportion. AAV vector samples with three different ssDNA lengths (1487, 2712, and 4133 bases) were used to analyze the genome mass under the MS conditions that can disassemble FP in the section of the transfer tube to flatapole. Their theoretical molecular weights were 460.2, 840.6, and 1279.6 kDa for 1487, 2712, and 4133 bases, respectively. To disassemble FP and analyze the molecular weight of the encapsidated genome, the IST voltage and spray voltage were set to −150 V and 1.8–2.2 kV, respectively.

### Analytical Ultracentrifugation

Size distribution analysis was performed by sedimentation velocity AUC (SV-AUC) and band sedimentation AUC (BS-AUC), in accordance with our previous study.^18,35^ SV-AUC was performed to determine the extinction coefficients of EP and FP, whereas BS-AUC was performed to evaluate the accuracy of relative quantitation and to assess the LORQ. Briefly, the AAV vector samples for AUC were diluted with the buffer to achieve FP concentrations of 4.0–9.0 × 10^12^ cp/mL, so that the absorbance of UV light at 230 nm was within the dynamic range. For SV-AUC, 390 μL of each EP or FP sample was loaded into the 12 mm double-sector charcoal-filled Epon centerpiece, and 400 μL of a corresponding solvent was loaded into each reference sector. Data were collected at 20 °C using the Optima AUC (Beckman Coulter, Brea, CA) at 20,000 rpm with UV detection at 230, 260, and 280 nm and an interference (IF) detection system. For BS-AUC, 15 μL of AAV vector sample was loaded into the sample reservoir well, and 15 μL of 1 × PBS with 0.001% poloxamer-188 and 200 mM NaCl was loaded into the reference reservoir well. A total of 240 or 250 μL of PBS/H_2_^18^O with 0.001% poloxamer-188 was loaded into the sample or reference sector, respectively. Data were collected at 20 °C using the Optima AUC at 20,000 rpm with a UV detection system at 230 nm. The collected data were analyzed with a continuous *c*(s) distribution model for SV-AUC and analytical zone centrifugation *c*(s) model for BS-AUC using SEDFIT (version 16.2b),^36^ where the lamella width, frictional ratio, meniscus, time-invariant noise, and radial-invariant noise were fitted, and a regularization level of 0.68 was used. The sedimentation coefficient range of 0–250 S was evaluated with a resolution of 500 for SV-AUC, while 0–175 S was evaluated with a resolution of 350 for BS-AUC. The extinction coefficients of EP and FP were determined as described previously.^35^*dn*/*dc* of AAV8 EP was calculated by SEDNTERP ver.3.

### Mass Photometry

Precision cover glasses (1.5H thickness, 24 × 50 mm; THORLABS, Newton, NJ) were cleaned with ethanol and Milli-Q water for 5 min each and dried with a blower, and then, the CultureWell gaskets (Grace Bio-Laboratories, Bend, OR) were attached. A few drops of ImmersolTM 581 F (ZEISS, Jena, Germany) were added to the objective lens, and the cover glass was placed with care taken to prevent the entry of any bubbles. Measurements were carried out using TwoMP (Refeyn, Oxford, UK). AAV vector samples at predetermined proportions were used to evaluate the accuracy of relative quantitation and to assess the LORQ. Prior to analyzing AAV vector particles, samples were diluted with 1 × PBS buffer 40 to 100 times and analyzed for 1 min three times each relative to their respective proportions. Every five-six analyses, BSA, apoferritin, and thyroglobulin (Sigma-Aldrich) were analyzed as calibrants.

### Capillary Gel Electrophoresis for ssDNA Analysis

The AAV vector samples were incubated with 3 μL of DNase and 1.5 μL of benzonase at 37 °C for 30 min to degrade any free DNA. The DNA digestion was inactivated with 10 μL of 50 mM EDTA, and simultaneously Proteinase K (QIAGEN, Hilden, Germany) was added at 20 mg/mL and incubated at 55 °C for 60 min. Subsequently, the sample was incubated at 95 °C for 20 min to digest the capsid and the lysate was collected by centrifugation. The collected lysate was purified, and electrophoresis was performed in a capillary filled with 5 mL of Nucleic Acid Extended Range Gel (SCIEX, Framingham, MA) containing 10 μL of SYBRTM GreenII RNA Gel Stain (SCIEX). DNA was measured using a PA800 Plus (SCIEX) instrument with 488 nm laser excitation fluorescence.

### Capillary Gel Electrophoresis for VP Component Analysis

AAV vector samples with a volume of 10 and 450 μL of buffer containing 0.5 mL of mercaptoethanol (Nacalai Tesque, Japan) and 9.5 mL of SDS (Nippon Gene Co., Ltd., Japan) were added to an Amicon Ultra −0.5–30 kDa (Merck Millipore Ltd., Germany) and centrifuged at 14,000*g* and 20 °C for 10 min twice. The filter was inverted onto a new collection tube and further centrifuged at 200*g* for 0.5 min. The filter was discarded, and the sample was incubated at 60 °C for 3 min. VP components were measured using PA800 Plus (SCIEX) with 214 nm laser-excited fluorescence.

## Results and Discussion

### Evaluation of the Accuracy of Relative Quantification and LORQ by Native CDMS

Prior to CDMS, SV-AUC was also carried out to confirm the purity of the EP 100% and FP 100% samples. According to previous studies,^35^ the main peaks at 65 and 91 S observed in each sample correspond to EP and FP, respectively (Figure S3). Since the intensity of the minor peaks in the results of both EP 100% and FP100% samples was below the LOQ as 0.0038. EP 100% and FP 100% samples are considered to contain 100% pure EP or FP, and the following calculations were conducted. Accuracy in relative quantification and LORQ was evaluated by analyzing the E/F ratio of AAV vectors at predetermined proportions of EP; because EP could be largely removed by purification, LORQ was evaluated under low-EP concentrations. In CDMS measurements, it should be noted that AAV vectors can be disassembled by thermal energy or the energy of gas collisions in the low-vacuum region. As shown in Figure S4(a), the disassembly of capsids by IST occurred, and the ease of capsid disassembly differed between EP and FP or was dependent on genome length. Additionally, as demonstrated in Figures S4(b) and S5, the capsids of FP and EP become VP fragments after disassembly; therefore, the disassembly of FP did not increase the absolute amount of EP in sample solution. Thus, we set the IST at −15 V, at which both EP and FP slightly or hardly disassembled for the evaluation of relative quantification.

**Figure 1:**
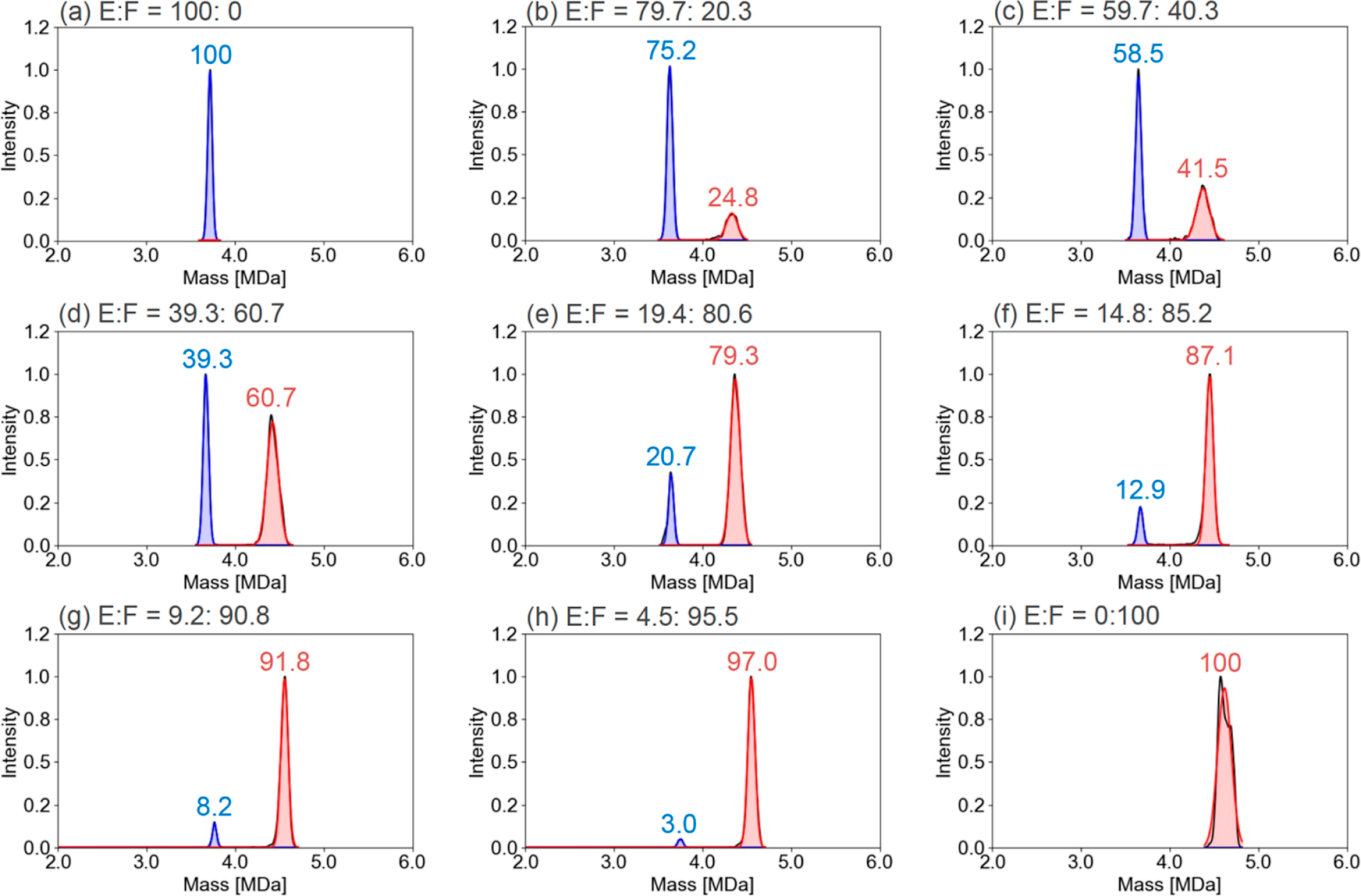
E/F ratio evaluation by CDMS. AAV vector samples with predetermined relative proportions of EP of (a–i) 100%, 79.7%, 59.7%, 39.3%, 19.4%, 14.8%, 9.2%, 4.5%, and 0% were used. The blue and red lines enclose the respective regions of EP and FP, calculated by Gaussian fitting. Measured proportions calculated with *N* = 3 are shown above each peak top.

**Figure 2:**
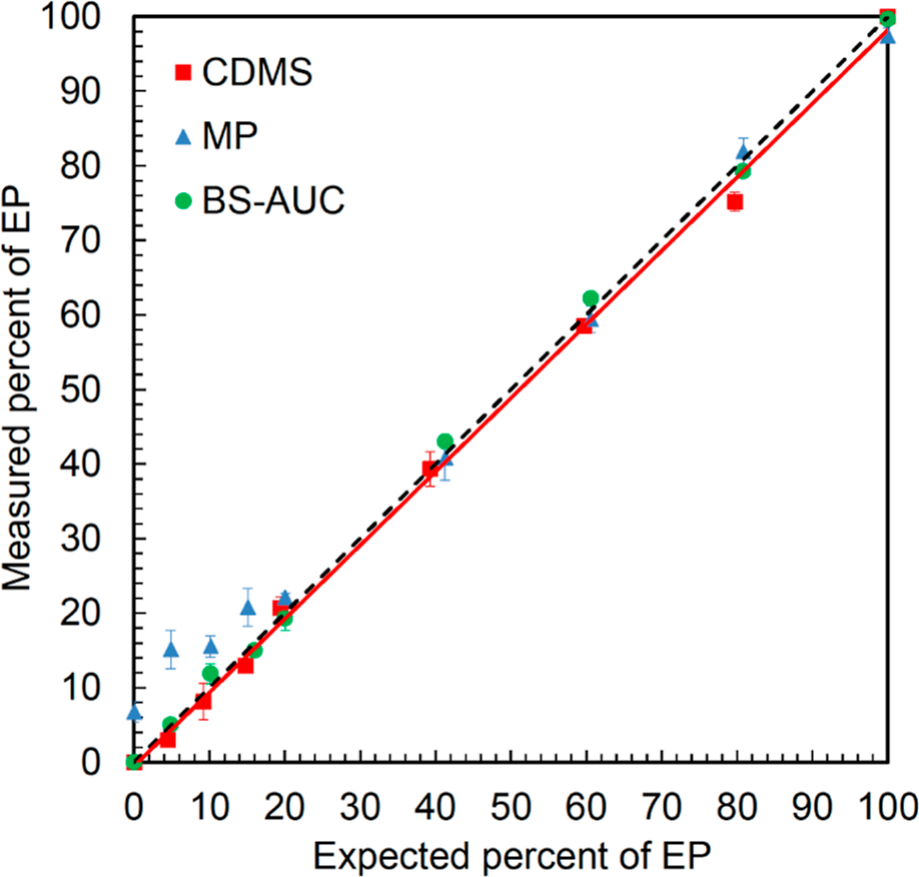
Linearity of E/F ratio quantification by CDMS, BS-AUC, and MP. Red, green, and blue plots represent the proportions of EP measured by CDMS, BS-AUC, and MP for each sample, respectively, and the red solid line represents the correlation lines of the red plots. Data were acquired with *N* = 3 to calculate the average value and the standard deviation. Black dashed line indicates *y* = *x*.

As shown in Figure 1a–i, samples with relative EP proportions of 100%, 79.7%, 59.7%, 39.3%, 19.4%, 14.8%, 9.2%, 4.5%, and 0% were quantified. The measured EP proportions were 100%, 75.2%, 58.5%, 39.3%, 20.7%, 12.9%, 8.2%, 3.0%, and 0%, respectively. In the experiment with EP of 4.5%, where the absolute amount of EP was low, at least 2000 ions were detected even after filtering using DMT analysis. The relative quantification results were within ±20% of the expected values up to EP 9.2%, confirming that the LORQ of EP by CDMS is 4.5–9.2%. In addition, it was found that EP could be detected even in samples with the expected EP proportion of 3.0%, as shown in Figure S6. Figure 2 shows the correlation (correlation coefficient *R* = 0.9988) between the expected EP proportion and the measured EP proportion for plot points, where accurate relative quantification was performed by CDMS. These results show that CDMS is a robust method for evaluating the E/F ratio of AAV vectors. Meanwhile, it was found that the results of relative quantification by mass spectrometry were underestimated when the absolute concentration of EP fall below the MS’s LOQ.

### Comparison of CDMS Results on E/F Ratio with Those from BS-AUC and MP on E/F Ratio

To compare the results of CDMS with the conventional methods in terms of accuracy and LORQ for the E/F ratio assessment, BS-AUC and MP were conducted. As shown in Figure 2, the relative quantification results were within ±20% of expected values with all predetermined proportions, confirming that the LORQ of EP by BS-AUC was 0–4.9%. The correlation coefficients for CDMS and BS-AUC were 0.9988 and 0.9993, respectively, indicating that there was no significant difference between CDMS and BS-AUC in the accuracy of evaluation of the E/F ratio when assessing proportions above the LORQ of CDMS. Meanwhile, as shown in Figure 2, the relative quantification results of MP were within ±20% of expected values up to 20.0% EP, confirming that the LORQ of EP by MP was 15.1–20.0%. The linear approximation for 20.0% or more showed a correlation coefficient of 0.9992, indicating that it has accuracy similar to that of CDMS and BS-AUC. The proportion of EP in MP was overestimated under the conditions with small EP proportions possibly due to the noise adjacent to the distribution of FP, as shown in Figure S7. Meanwhile, the data acquisition time for MP analysis was only 1 min, which was significantly shorter than ∼50 min for CDMS and ∼2.5 h for BS-AUC. These results suggest that CDMS, BS-AUC, and MP are orthogonal methods for E/F ratio evaluation.

### Encapsidated Genome Analysis Due to Capsid Disassembly within Mass Spectrometer by Dissociation CDMS

**Figure 3:**
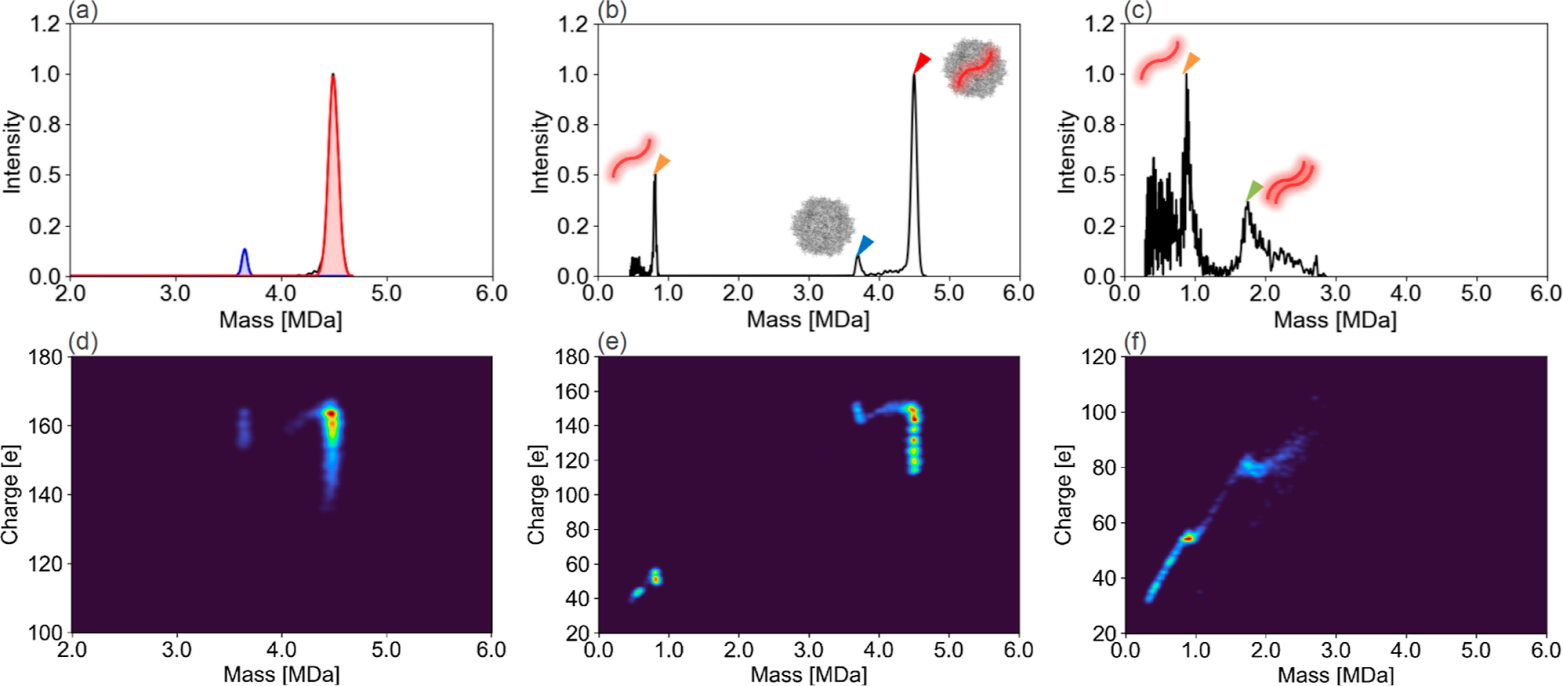
Evaluation of the E/F ratio and analyses of encapsidated genome by orbitrap-based CDMS. Direct analysis using CDMS on AAV8-FIXp encapsidating genome of 2712 bases was performed. (a) Areas enclosed by blue and red lines represent the regions of EP and FP, respectively. The E/F ratio was estimated to be 6.8%. (b) Orange, blue, and red arrowheads indicate the peaks of genome, EP, and FP, respectively. The genome mass was 820.7 kDa (the theoretical mass is 840.6 kDa). (c) CDMS analysis of DNA extracted by proteinase K treatment from AAV8-FIXp. The orange and green arrowheads indicate the ssDNA, originally encapsidated in the AAV8-FIXp and the dsDNA formed by annealing of ssDNA during the genome extraction process, respectively. The heat maps of the charge vs mass obtained by CDMS corresponding to (a–c) are shown in (d–f), respectively.

As part of the multimass analysis, determination of encapsidated genome mass was conducted by CDMS of three different AAV vectors (AAV8-H4C1, AAV8-FIXp, and AAV8-FIXp hFIX mini-intron), which have genome length of 1487, 2712, and 4133 bases, respectively. Figure 3a,d shows that the mass and charge distribution of AAV8-FIXp acquired by CDMS could separate peaks according to genome packaging status. The peak at 3.71 MDa corresponded to EP with a theoretical value of 3.75 MDa, and the 4.50 MDa peak corresponded to FP with a theoretical value of 4.60 MDa. AAV vectors can be disassembled by thermal energy or the energy of gas collisions in the low-vacuum region. By intentionally dissociating FP in the low-vacuum region with high IST at −150 V, a new peak was observed at 820.7 kDa in addition to EP and FP peaks, as shown in Figure 3b,e. Since the theoretical genome mass of 2712 bases is 840.6 kDa, the new peak is considered to correspond to the encapsidated genome in FP of AAV8-FIXp, with errors of 2.4% (2.4 × 10^4^ ppm) compared with the theoretical value. No new peak appeared when analyzing EPs under the same high IST conditions, as shown in Figure S5, which suggested that the new peak may represent the encapsidated genome. Thus, the components with lower mass than the encapsidated genome shown in Figure 3b,c were considered to be the complexes of viral protein and fragmented DNA. We also subjected AAV8-FIXp to genome extraction treatment using proteinase K at 55 °C for 180 min and analyzed the extracted genome by CDMS by the same method, as shown in Figure 3c,f. At this time, the extracted genome showed the same mass as when the capsid disassembly occurred within the mass spectrometer, as indicated by the orange arrowhead in Figure 3b,c. These results demonstrated that CDMS could directly analyze the mass of the genome encapsidated in the AAV vectors without pretreatment. Additionally, in Figure 3c, there is a peak with approximately twice the mass (1752 kDa) of the genome indicated by the green arrowhead. Given that the genome length of FIXp’s ssDNA is about 2.7 kb and that the genome encapsidated by AAV can be up to 4.7 kb, this peak is considered to be dsDNA resulting from the annealing of ssDNA during the pretreatment process. A previous study^30^ also revealed that dsDNA can be detected in the CDMS analysis of extracted genomes that have undergone pretreatment processes such as heating or enzyme treatment. Another previous study^37^ that analyzed the genome extracted from AAV using MP only detected peaks of ssDNA. This is likely attributable to the short duration of the heating treatment, which was insufficient for annealing to occur. These findings suggest that encapsidated genome analysis by disassembly inside the mass spectrometer, which does not require pretreatment, allows more accurate analysis of the genuinely encapsidated genome as it avoids structural changes, including denaturation and annealing, caused by pretreatment.

**Figure 4:**
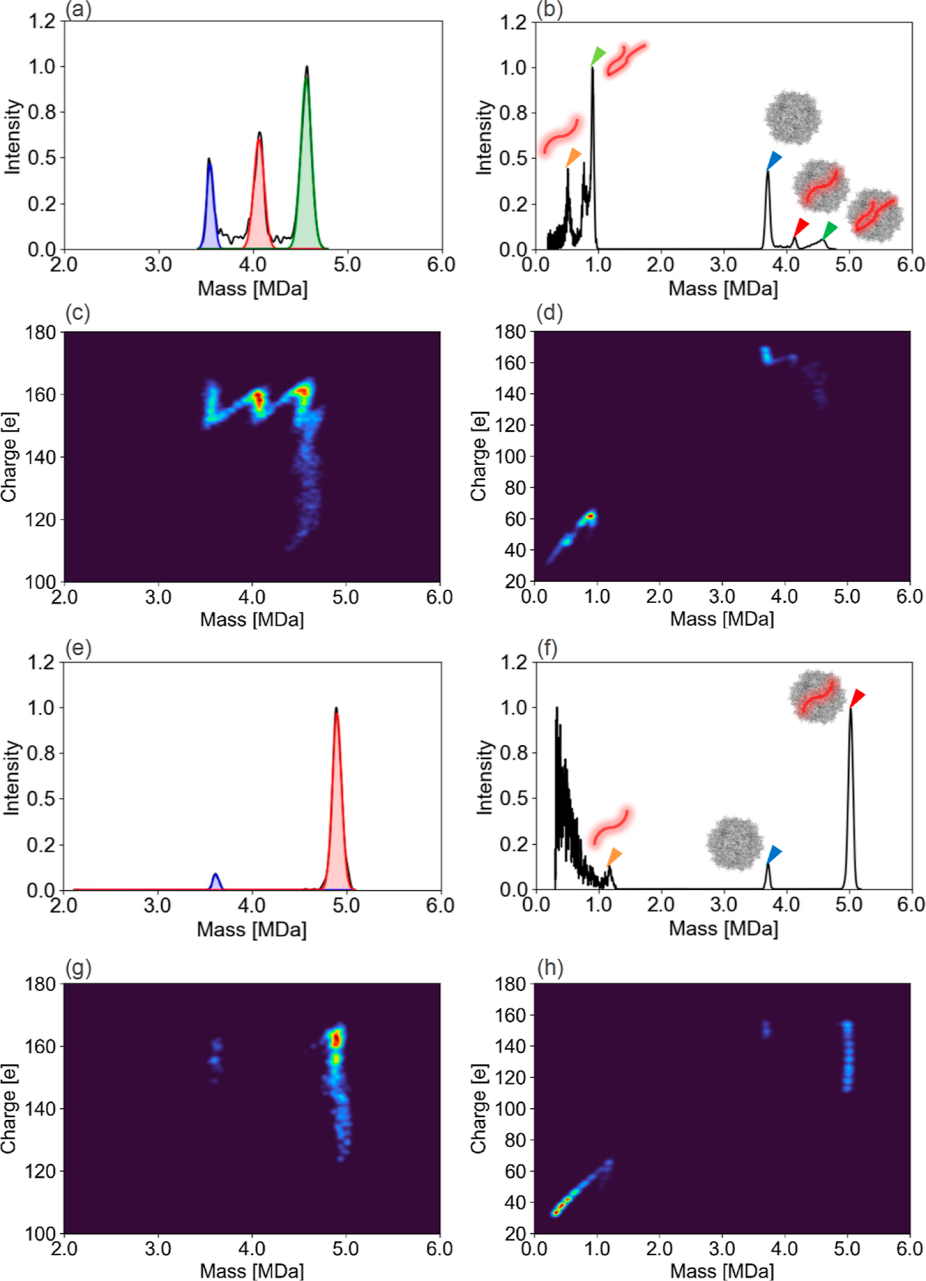
Evaluation of the E/F ratio and analyses of encapsidated genome using CDMS. Direct analysis using CDMS on AAV8-H4C1 and AAV8-FIXp hFIX mini-intron encapsidating genome of 1487 and 4133 bases was performed. (a and e) Areas enclosed by the blue, red, and green lines represent the regions of EP, FP, and OP, respectively. From capsid content distribution, the E/F ratios (or EP and OP to FP ratio) were 68.2% and 5.5%. (b and f) Orange, light green, blue, red, and green arrowheads indicate the peaks of ssDNA, dsDNA (or self-complementary DNA), EP, FP, and OP, respectively. The genome masses of ssDNA were (b) 515.8 and (f) 1170.7 kDa. Heat maps of the charge vs mass obtained by CDMS corresponding to (a, b, e, and f) are shown in (c, d, g, and h), respectively.

Figure 4 shows the integrity by native CDMS and direct genome evaluation by dissociation CDMS analysis for AAV8-H4C1 and AAV8-FIXp hFIX mini-intron. Figure 4a shows that the AAV8-H4C1 sample contains OP. The mass difference between EP and OP allows us to calculate that the encapsidated genome in OP is 985 kDa, corresponding to the green arrowhead in Figure 4b. The mass of the genome encapsidated in OP corresponds to the 3234 bases shown in Figure S8(a), representing dsDNA or 3ITR+2GOI.^38^Figure 4b,f shows that the encapsidated ssDNA were 518.5 and 1170.7 kDa, with errors of 12.6% (1.3 × 10^5^ ppm) and 8.5% (8.5 × 10^4^ ppm), respectively, compared with the theoretical values of 460.2 and 1279.6 kDa. The encapsidated genomes of 460.2, 820.7, and 1279.6 kDa analyzed by dissociation CDMS correspond to the 1202, 2988, and 3924 bases obtained by CE-LIF, as shown in Figure S8. The genome lengths obtained by CE-LIF differed by 19.2%, 10.2%, and 5.1% from the theoretical lengths of 1487, 2712, and 4133 bases, respectively.

These results indicate that CDMS possesses the precision for encapsidated genome analysis and can be used as a method orthogonal to CE-LIF. However, when sequence information is required, next-generation sequencers^39−41^ should be used. We also conducted encapsidated genome analysis using AAV5-CMV-EGFP, where AAV5 is known for its higher capsid stability than other serotypes,^42^ as shown in Figure S9. The peak corresponding to the encapsidated genome was observed, demonstrating that genome analysis through capsid disassembly is applicable even for AAVs with the high capsid stability.

The lower mass accuracy of DNA may be due to changes in DNA mass caused by methylation, adduct ions, and the addition of proteins derived from VP. Additionally, the structural instability of DNA during orbital motion in the analyzer section could potentially impact the mass accuracy. It was suggested from experiments using ion-trap-based CDMS that the charge carried by DNA may change depending on its folding state.^43^ Another study suggested that the desolvation or charge stripping of the ions being analyzed within the Orbitrap analyzer can shift the mass.^44^ Therefore, it is conceivable that changes in the folding state of DNA during orbital motion in the analyzer section could lead to mass or charge uncertainty. The high-precision analysis of the genome encapsidated in AAV8-FIXp suggests that the accuracy of DNA analysis by CDMS could be improved with further optimization of the ion optic conditions.

### Estimation of VP Stoichiometric Variation from Mass Distribution by CDMS

**Figure 5:**
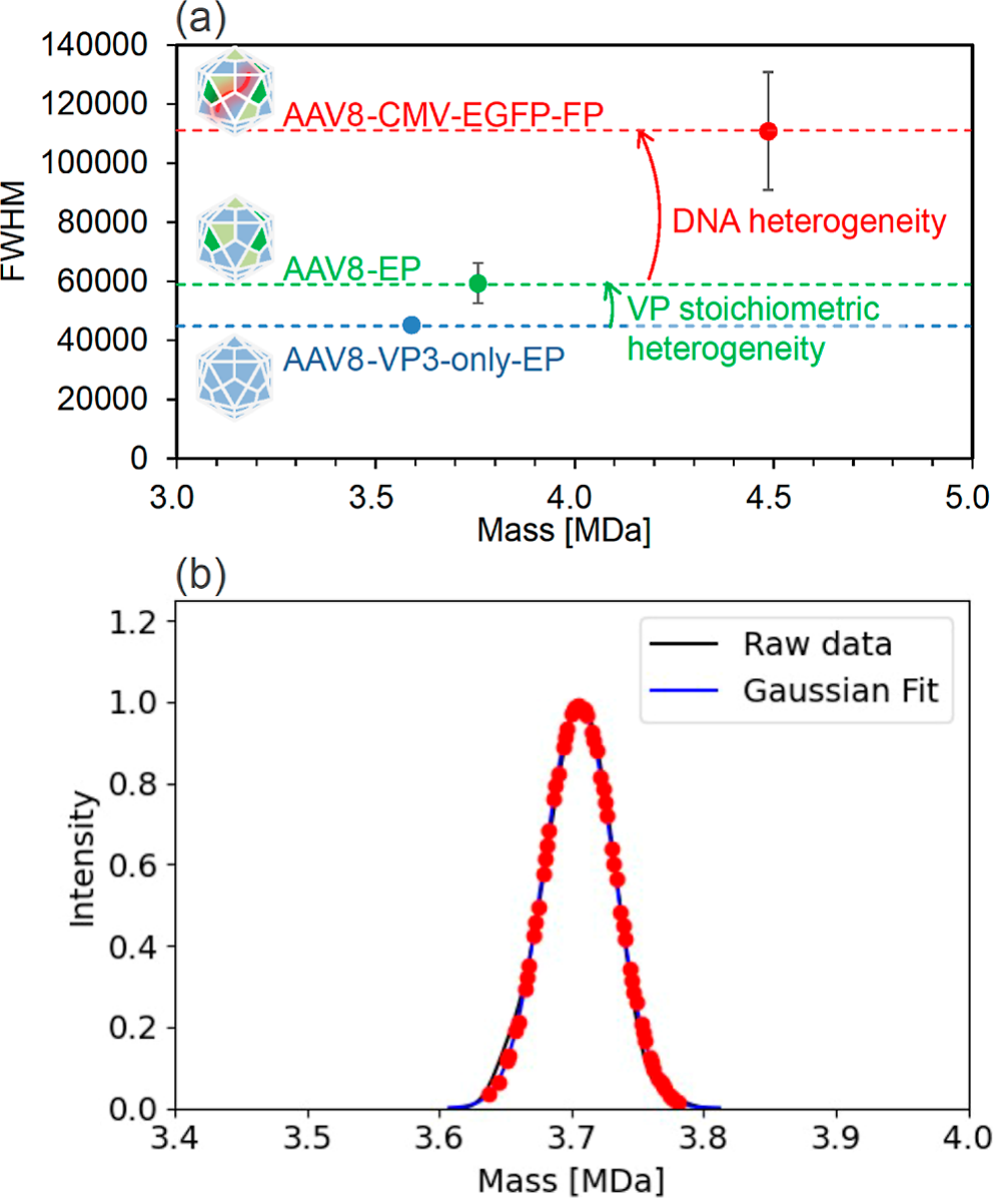
Estimation of VP stoichiometry using the correlation between mass distribution obtained by CDMS and AAV vector heterogeneity. (a) Differences in the fwhm of AAV8-VP3-only-EP, AAV8-EP, and AAV8-CMV-EGFP-FP suggest that VP stoichiometry and encapsidated genome contribute to the heterogeneity in the viral particles. (b) Mass distribution of AAV8-FIXp hFIX mini-intron-EP was fitted with a Gaussian function, and mass values that can be adopted at integer stoichiometry of VP are plotted as red points.

As part of the multimass analysis, estimation of VP stoichiometry was also attempted using the data acquired by CDMS. The spectra of AAV vectors were broad due to the diversity in their charge, VP composition,^45^ isotopes, adducts, and encapsidated genome. There were differences in the full width at half-maximum (fwhm), comparing AAV8-VP3-only-EP (used in charge calibration), AAV8-EP, and AAV8-CMV-EGFP-FP, as shown in Figure 5a. This suggests that the width of spectra obtained from mass spectrometry reflects the heterogeneity of AAV vectors. A difference in fwhm between AAV8-VP3-only-EP (4.5 × 10^4^) and AAV8-EP (5.9 × 10^4^) reflects stoichiometric heterogeneity of VP. Additionally, a difference in fwhm between AAV8-EP and AAV8-CMV-EGFP-FP (1.1 × 10^5^) reflects DNA heterogeneity, length, polymorphism, or tiny fragments and stoichiometric heterogeneity of VP.

Therefore, we checked whether the obtained mass distribution could have a value that VP1, VP2, and VP3 can adopt in an integer stoichiometry using Python (the algorithm is described in the Supporting Information). Red points in Figure 5b show mass values that can be adopted at the VP integer stoichiometry on the mass distribution. In other words, this mass distribution obtained by CDMS indicates that the analyzed AAV vectors are a collection of capsids with VP ratios assigned by the red points. The averaged stoichiometry of VP was calculated by weighted averaging based on these intensity values. From the results, the average stoichiometry of EP capsids, VP1: VP2: VP3, of AAV8-H4C1, AAV8-FIXp, and AAV8-FIXp hFIX mini-intron was estimated to be 2.6:9.4:48.0, 2.6:9.4:48.0, and 2.5:9.4:48.0, respectively. In this calculation, limitations were set at 0–60 for each estimated stoichiometry of VP1, VP2, and VP3. The similarity in the estimated VP ratios arises because the mass distributions of the EPs determined by CDMS are consistent with each other.

Furthermore, by subtracting the mass of the genome from the mass distribution of FP obtained in Figure 4 and conducting the analysis for the capsid of FP, we estimated the VP stoichiometry of AAV8-H4C1, AAV8-FIXp, and AAV8-FIXp hFIX mini-intron to be 1.8:7.1:51.0, 1.7:6.6:51.7, and 3.4:12.3:44.2, respectively. The difference in the stoichiometry between EP and capsids of FP is primarily considered to be due to the encapsidation of DNA. Moreover, these results suggest the possibility that the VP stoichiometry depends on the presence and length of the encapsidated genome in the AAV vectors. For AAV8-H4C1, AAV8-FIXp, and AAV8-FIXp hFIX mini-intron, the VP ratios measured by CE-SDS were 5.1:7.8:47.0, 4.6:9.3:46.1, and 4.7:7.5:47.8, respectively, as shown in Figure S10. The method for estimating VP stoichiometry assumes that the mass distribution obtained by CDMS fully reflects the VP stoichiometry. In practice, the mass distribution can be broadened or shifted due to heterogeneity originating from the instrument or the sample, and the mass values themselves can vary due to the charge accuracy of CDMS. These factors most likely explain why the estimated stoichiometry differs from the results obtained by CE-SDS. Meanwhile, despite the wide range of possible values for VP1, VP2, and VP3 (ranging from 0 to 60), the estimated values do not significantly differ from those obtained by CE-SDS. This indicates that CDMS is a viable method for estimating VP stoichiometry and can be used as a method orthogonal to CE-SDS.

### Results of Multimass Analysis by Orbitrap-Based CDMS and Comparison with Conventional Methods

Table 1 shows a summary of AAV vector characterization of AAV8-H4C1, AAV8-FIXp, and AAV8-FIXp hFIX mini-intron by CDMS, BS-AUC, MP, CE-LIF, and CE-SDS. The results of integrity evaluation by BS-AUC and MP are shown in Figure S11. While CDMS and BS-AUC demonstrate similar relative quantification results for all AAV vectors, the lower resolution of MP makes it difficult to separate EP, FP, and OP when the genome size is small, and it also leads to the overestimation of EP due to noise near FP. These findings are well-reproduced in the results shown in Figure 2 using AAV8-CMV-EGFP, reaffirming that CDMS and BS-AUC are robust methods for the relative quantification of EP. Importantly, CDMS allows the multimass analysis of AAV vectors, that is, multiple evaluations of EP mass, FP mass, encapsidated genome mass, E/F ratio, and VP stoichiometry.

**Table 1:** Multimass Analysis by Orbitrap-Based CDMS

AAV vectors	quality attributes	theoretical value	CDMS	BS-AUC	MP	CE-LIF	CE-SDS^a^
AAV8-H4C1	EP mass [MDa]	3.73	3.71		3.71		
	FP mass [MDa]	4.19	4.13		4.17		
	ssDNA mass/length [kDa]/[bases]	460.2/1487	518.4 kDa			1202 bases	
	EP + OP to FP ratio [%]		68.2	63.4	NA		
	VP stoichiometry VP1: VP2: VP3	EP		2.6:9.4:48.0			5.1:7.8:47.0^b^
		capsid of FP		1.8:7.1:51.0			
AAV8-FIXp	EP mass [MDa]	3.75	3.71		NA		
	FP mass [MDa]	4.60	4.50		4.42		
	ssDNA mass/length [kDa]/[bases]	840.6/2712	820.7 kDa			2988 bases	
	E/F ratio [%]		6.8	6.4	13.1		
	VP stoichiometry VP1: VP2: VP3	EP		2.6:9.4:48.0			4.9:9.7:45.4
		capsid of FP		1.7:6.6:51.7			4.6:9.3:46.1
AAV8-FIXp hFIX-mini-intron	EP mass [MDa]	3.74	3.71		NA		
	FP mass [MDa]	5.02	5.03		4.81		
	ssDNA mass/length [kDa]/[bases]	1279.6/4133	1170.7 kDa			3924 bases	
	E/F ratio [%]		5.5	4.8	8.3		
	VP stoichiometry VP1: VP2: VP3	EP		2.5:9.4:48.0			5.0:9.6:45.4
		capsid of FP		3.4:12.3:44.2			4.7:7.5:47.8

a VP3 ratio represents the sum of VP3 and VP3 clip.

b As AAV8-H4C1 was not purified and separated into EP and FP by DG-UC, the CE-SDS result gave a ratio of these mixtures.

## Conclusions

In this study, we elucidated the accuracy and LORQ of the assessment of E/F ratio by CDMS, in comparison to BS-AUC and MP. The results showed that CDMS is a method capable of evaluating the E/F ratio under low concentration conditions, comparable to BS-AUC. Ion loss occurs at each section in the mass spectrometer, and there is a limit to the ions that can reach the analyzer section. As a result, the E/F ratios of CDMS analysis under conditions with a low proportion of EP are slightly underestimated. However, it was found that CDMS has a detection capacity even when the relative proportion of EP is as low as 3.0%. These results indicate that CDMS is a viable way to assess the E/F ratio.

Multimass analysis on the three different AAV vectors was conducted with comparisons with each method, BS-AUC, MP, CE-LIF, and CE-SDS. CDMS can accurately distinguish peaks according to packaging status with the mass of each AAV particle and directly analyze the mass of the encapsidated genome without pretreatment. A comparison with CE-LIF showed agreement with regard to the size of the genome encapsidated in the AAV vectors. However, the average accuracy in the full-length analysis of the ssDNA by CDMS and CE-LIF was found to be 7.8% and 11.5%, respectively, revealing the possibility of further optimization of the analytical conditions for the encapsidated genome. Furthermore, by comparing fwhm values of AAV8-VP3-only-EP and AAV8-EP, it was discovered that the mass distribution obtained by CDMS reflects the heterogeneity of AAV vectors. The estimation of the averaged stoichiometry of VP was also successfully accomplished by assigning the mass values of the mass distribution represented by the VP integer stoichiometry.

It was thus revealed that CDMS is not only a powerful mass spectrometric approach for high-molecular-weight samples, such as viral vectors, with high heterogeneity but can also be applied to the assessment of multiple characteristics, including E/F ratio, encapsidated genome, and VP stoichiometry. In the assessments of genome and VP stoichiometry, CDMS has demonstrated an accuracy equal to or greater than that of the existing standard methods. Multimass analysis by orbitrap-based CDMS provides data complementary to conventional methods, and with further improvement in mass and charge accuracy, it has the potential to serve as a replacement of each of these methods. To achieve a comprehensive analysis of AAV vectors using CDMS, it will be necessary to detect protein and DNA impurities with higher resolving power. This requires further improvements in the charge accuracy of the CDMS.

## Supporting Information Available

CE-SDS analysis of AAV8-VP3-only-EP; charge calibration for Orbitrap-CDMS analysis; SV-AUC assessment for EP 100% and FP 100% samples; dependency of AAV capsid disassembly on in-source trapping voltage; EP disassemble by dissociation CDMS; CDMS analysis of EP 3% sample; noise components during MP analysis; CE-LIF analysis for encapsidated genome length of AAV8-H4C1, AAV8-FIXp, and AAV8-FIXp hFIX mini-intron; evaluation of encapsidated genome in AAV5-CMV-EGFP; VP ratio evaluation by CE-SDS; integrity evaluation of AAV8-H4C1, AAV8-FIXp, and AAV8-FIXp hFIX mini-intron by BS-AUC and MP; and VP estimation algorithm (PDF)

## References

[ref1] Wang J. H.; Gessler D. J.; Zhan W.; Gallagher T. L.; Gao G. Adeno-Associated Virus as a Delivery Vector for Gene Therapy of Human Diseases. Signal Transduction Targeted Ther. 2024, 9, 78 10.1038/s41392-024-01780-w. PMC10987683 38565561

[ref2] Wang D.; Tai P. W. L.; Gao G. Adeno-Associated Virus Vector as a Platform for Gene Therapy Delivery. Nat. Rev. Drug Discovery 2019, 18, 358–378. 10.1038/s41573-019-0012-9. 30710128 PMC6927556

[ref3] Johnson F. B.; Ozer H. L.; Hoggan M. D. Structural Proteins of Adenovirus-Associated Virus Type 3. J. Virol. 1971, 8 (6), 860–863. 10.1128/jvi.8.6.860-863.1971. 5172922 PMC376276

[ref4] Buller R. M. L.; Rose J. A. Characterization of Adenovirus-Associated Virus-Induced Polypeptides in KB Cells. J. Virol. 1978, 25, 331–338. 10.1128/jvi.25.1.331-338.1978. 621779 PMC353931

[ref5] Sha S.; Maloney A. J.; Katsikis G.; Nguyen T. N. T.; Neufeld C.; Wolfrum J.; Barone P. W.; Springs S. L.; Manalis S. R.; Sinskey A. J.; Braatz R. D. Cellular Pathways of Recombinant Adeno-Associated Virus Production for Gene Therapy. Biotechnol. Adv. 2021, 49, 107764 10.1016/j.biotechadv.2021.107764. 33957276

[ref6] Wu Z.; Asokan A.; Samulski R. J. Adeno-Associated Virus Serotypes: Vector Toolkit for Human Gene Therapy. Mol. Ther. 2006, 14, 316–327. 10.1016/j.ymthe.2006.05.009. 16824801

[ref7] Hebben M. Downstream Bioprocessing of AAV Vectors: Industrial Challenges & Regulatory Requirements. Cell Gene Ther Insights 2018, 4 (2), 131–146. 10.18609/cgti.2018.016.

[ref8] Terova O.; Soltys S.; Hermans P.; De Rooij J.; Detmers F. Overcoming Downstream Purification Challenges for Viral Vector Manufacturing: Enabling Advancement of Gene Therapies in the Clinic. Cell Gene Ther Insights 2018, 4 (2), 101–111. 10.18609/cgti.2018.017.

[ref9] Gao K.; Li M.; Zhong L.; Su Q.; Li J.; Li S.; He R.; Zhang Y.; Hendricks G.; Wang J.; Gao G. Empty Virions in AAV8 Vector Preparations Reduce Transduction Efficiency and May Cause Total Viral Particle Dose-Limiting Side Effects. Mol. Ther.--Methods Clin. Dev. 2014, 1, 20139 10.1038/mtm.2013.9. 25485285 PMC4255953

[ref10] Pei X.; Earley L. F.; He Y.; Chen X.; Hall N. E.; Samulski R. J.; Li C. Efficient Capsid Antigen Presentation from Adeno-Associated Virus Empty Virions in Vivo. Front. Immunol. 2018, 9, 844 10.3389/fimmu.2018.00844. 29725339 PMC5916967

[ref11] Onishi T.; Nonaka M.; Maruno T.; Yamaguchi Y.; Fukuhara M.; Torisu T.; Maeda M.; Abbatiello S.; Haris A.; Richardson K.; et al. Enhancement of Recombinant Adeno-Associated Virus Activity by Improved Stoichiometry and Homogeneity of Capsid Protein Assembly. Mol. Ther.--Methods Clin. Dev. 2023, 31, 101142 10.1016/j.omtm.2023.101142. 38027055 PMC10663676

[ref12] Popa-Wagner R.; Porwal M.; Kann M.; Reuss M.; Weimer M.; Florin L.; Kleinschmidt J. A. Impact of VP1-Specific Protein Sequence Motifs on Adeno-Associated Virus Type 2 Intracellular Trafficking and Nuclear Entry. J. Virol. 2012, 86 (17), 9163–9174. 10.1128/JVI.00282-12. 22696661 PMC3416132

[ref13] Xiao P.-J.; Samulski R. J. Cytoplasmic Trafficking Endosomal Escape, and Perinuclear Accumulation of Adeno-Associated Virus Type 2 Particles Are Facilitated by Microtubule Network. J. Virol. 2012, 86 (19), 10462–10473. 10.1128/jvi.00935-12. 22811523 PMC3457265

[ref14] Wright J. F. Quality Control Testing, Characterization and Critical Quality Attributes of Adeno-Associated Virus Vectors Used for Human Gene Therapy. Biotechnol. J. 2021, 16 (1), 2000022 10.1002/biot.202000022. 33146911

[ref15] Dorange F.; Le Bec C. Analytical Approaches to Characterize AAV Vector Production & Purification: Advances and Challenges. Cell Gene Ther Insights 2018, 4 (2), 119–129. 10.18609/cgti.2018.015.

[ref16] Werle A. K.; Powers T. W.; Zobel J. F.; Wappelhorst C. N.; Jarrold M. F.; Lyktey N. A.; Sloan C. D. K.; Wolf A. J.; Adams-Hall S.; Baldus P.; et al. Comparison of Analytical Techniques to Quantitate the Capsid Content of Adeno-Associated Viral Vectors. Mol. Ther.--Methods Clin. Dev. 2021, 23, 254–262. 10.1016/j.omtm.2021.08.009. 34703846 PMC8505359

[ref17] Hirohata K.; Yamaguchi Y.; Maruno T.; Shibuya R.; Torisu T.; Onishi T.; Chono H.; Mineno J.; Yuzhe Y.; Higashiyama K.; et al. Applications and Limitations of Equilibrium Density Gradient Analytical Ultracentrifugation for the Quantitative Characterization of Adeno-Associated Virus Vectors. Anal. Chem. 2024, 96, 642–651. 10.1021/acs.analchem.3c01955. 38165078 PMC10794998

[ref18] Maruno T.; Ishii K.; Torisu T.; Uchiyama S. Size Distribution Analysis of the Adeno-Associated Virus Vector by the c (s) Analysis of Band Sedimentation Analytical Ultracentrifugation with Multiwavelength Detection. J. Pharm. Sci. 2023, 112 (4), 937–946. 10.1016/j.xphs.2022.10.023. 36374763

[ref19] Wagner C.; Fuchsberger F. F.; Innthaler B.; Lemmerer M.; Birner-Gruenberger R. Quantification of Empty, Partially Filled and Full Adeno-Associated Virus Vectors Using Mass Photometry. Int. J. Mol. Sci. 2023, 24 (13), 11033 10.3390/ijms241311033. 37446211 PMC10341871

[ref20] Barnes L. F.; Draper B. E.; Chen Y. T.; Powers T. W.; Jarrold M. F. Quantitative Analysis of Genome Packaging in Recombinant AAV Vectors by Charge Detection Mass Spectrometry. Mol. Ther.--Methods Clin. Dev. 2021, 23, 87–97. 10.1016/j.omtm.2021.08.002. 34631929 PMC8476707

[ref21] Pierson E. E.; Keifer D. Z.; Asokan A.; Jarrold M. F. Resolving Adeno-Associated Viral Particle Diversity with Charge Detection Mass Spectrometry. Anal. Chem. 2016, 88 (13), 6718–6725. 10.1021/acs.analchem.6b00883. 27310298 PMC6537880

[ref22] Ebberink E. H. T. M.; Ruisinger A.; Nuebel M.; Thomann M.; Heck A. J. R. Assessing Production Variability in Empty and Filled Adeno-Associated Viruses by Single Molecule Mass Analyses. Mol. Ther.--Methods Clin. Dev. 2022, 27, 491–501. 10.1016/j.omtm.2022.11.003. 36458114 PMC9706604

[ref23] Wörner T. P.; Snijder J.; Friese O.; Powers T.; Heck A. J. R. Assessment of Genome Packaging in AAVs Using Orbitrap-Based Charge-Detection Mass Spectrometry. Mol. Ther.--Methods Clin. Dev. 2022, 24, 40–47. 10.1016/j.omtm.2021.11.013. 34977271 PMC8671526

[ref24] Gustafson E. L.; Murray H. V.; Caldwell T.; Austin D. E. Accurately Mapping Image Charge and Calibrating Ion Velocity in Charge Detection Mass Spectrometry. J. Am. Soc. Mass Spectrom. 2020, 31 (10), 2161–2170. 10.1021/jasms.0c00263. 32856905

[ref25] Kafader J. O.; Beu S. C.; Early B. P.; Melani R. D.; Durbin K. R.; Zabrouskov V.; Makarov A. A.; Maze J. T.; Shinholt D. L.; Yip P. F.; et al. STORI Plots Enable Accurate Tracking of Individual Ion Signals. J. Am. Soc. Mass Spectrom. 2019, 30, 2200–2203. 10.1007/s13361-019-02309-0. 31512223 PMC6852666

[ref26] Kafader J. O.; Melani R. D.; Durbin K. R.; Ikwuagwu B.; Early B. P.; Fellers R. T.; Beu S. C.; Zabrouskov V.; Makarov A. A.; Maze J. T.; et al. Multiplexed mass spectrometry of individual ions improves measurement of proteoforms and their complexes. Nat. Methods 2020, 17 (4), 391–394. 10.1038/s41592-020-0764-5. 32123391 PMC7131870

[ref27] Wörner T. P.; Snijder J.; Bennett A.; Agbandje-McKenna M.; Makarov A. A.; Heck A. J. R. Resolving Heterogeneous Macromolecular Assemblies by Orbitrap-Based Single-Particle Charge Detection Mass Spectrometry. Nat. Methods 2020, 17 (4), 395–398. 10.1038/s41592-020-0770-7. 32152501

[ref28] Xiang Z.; Kurupati R. K.; Li Y.; Kuranda K.; Zhou X.; Mingozzi F.; High K. A.; Ertl H. C. J. The Effect of CpG Sequences on Capsid-Specific CD8+ T Cell Responses to AAV Vector Gene Transfer. Mol. Ther. 2020, 28 (3), 771–783. 10.1016/j.ymthe.2019.11.014. 31839483 PMC7054717

[ref29] Oyama H.; Ishii K.; Maruno T.; Torisu T.; Uchiyama S. Characterization of Adeno-Associated Virus Capsid Proteins with Two Types of VP3-Related Components by Capillary Gel Electrophoresis and Mass Spectrometry. Hum. Gene Ther. 2021, 32 (21–22), 1403–1416. 10.1089/hum.2021.009. 34082578 PMC10112878

[ref30] Barnes L. F.; Draper B. E.; Kurian J.; Chen Y.-T.; Shapkina T.; Powers T. W.; Jarrold M. F. Analysis of AAV-Extracted DNA by Charge Detection Mass Spectrometry Reveals Genome Truncations. Anal. Chem. 2023, 95, 4310–4316. 10.1021/acs.analchem.2c04234. 36880264

[ref31] Warrington K. H.; Gorbatyuk O. S.; Harrison J. K.; Opie S. R.; Zolotukhin S.; Muzyczka N. Adeno-Associated Virus Type 2 VP2 Capsid Protein Is Nonessential and Can Tolerate Large Peptide Insertions at Its N Terminus. J. Virol. 2004, 78 (12), 6595–6609. 10.1128/JVI.78.12.6595-6609.2004. 15163751 PMC416546

[ref32] Mietzsch M.; Liu W.; Ma K.; Bennett A.; Nelson A. R.; Gliwa K.; Chipman P.; Fu X.; Bechler S.; McKenna R.; et al. Production and Characterization of an AAV1-VP3 Only Capsid: An Analytical Benchmark Standard. Mol. Ther.--Methods Clin. Dev. 2023, 29, 460–472. 10.1016/j.omtm.2023.05.002. 37273903 PMC10238842

[ref33] Wu D.; Hwang P.; Li T.; Piszczek G. Rapid Characterization of Adeno-Associated Virus (AAV) Gene Therapy Vectors by Mass Photometry. Gene Ther. 2022, 29 (12), 691–697. 10.1038/s41434-021-00311-4. 35046529 PMC9296698

[ref34] Sommer J. M.; Smith P. H.; Parthasarathy S.; Isaacs J.; Vijay S.; Kieran J.; Powell S. K.; McClelland A.; Wright J. Quantification of Adeno-Associated Virus Particles and Empty Capsids by Optical Density Measurement. Mol. Ther. 2003, 7 (1), 122–128. 10.1016/s1525-0016(02)00019-9. 12573625

[ref35] Maruno T.; Usami K.; Ishii K.; Torisu T.; Uchiyama S. Comprehensive Size Distribution and Composition Analysis of Adeno-Associated Virus Vector by Multiwavelength Sedimentation Velocity Analytical Ultracentrifugation. J. Pharm. Sci. 2021, 110 (10), 3375–3384. 10.1016/j.xphs.2021.06.031. 34186069

[ref36] Schuck P. Sedimentation Analysis of Noninteracting and Self-Associating Solutes Using Numerical Solutions to the Lamm Equation. Biophys. J. 1998, 75 (3), 1503–1512. 10.1016/S0006-3495(98)74069-X. 9726952 PMC1299825

[ref37] Ebberink E. H. T. M.; Ruisinger A.; Nuebel M.; Meyer-Berg H.; Ferreira I. R. S.; Thomann M.; Heck A. J. R. Probing Recombinant AAV Capsid Integrity and Genome Release under Thermal Stress by Single-Molecule Interferometric Scattering Microscopy. bioRxiv 2024, 10.1101/2024.03.07.583968. PMC11295964 39100914

[ref38] Li X.; Miller L. M.; Chrzanowski M.; Tian J.; Jarrold M. F.; Herzog R. W.; Xiao W.; Draper B.; Zhang J. Quantitative Analysis of Preferential Utilization of AAV ITR as the Packaging Terminal Signal. Front. Bioeng. Biotechnol. 2023, 11, 1327433 10.3389/fbioe.2023.1327433. 38173872 PMC10761532

[ref39] Tran N. T.; Heiner C.; Weber K.; Weiand M.; Wilmot D.; Xie J.; Wang D.; Brown A.; Manokaran S.; Su Q.; Zapp M. L.; Gao G.; Tai P. W. L. AAV-Genome Population Sequencing of Vectors Packaging CRISPR Components Reveals Design-Influenced Heterogeneity. Mol. Ther.--Methods Clin. Dev. 2020, 18, 639–651. 10.1016/j.omtm.2020.07.007. 32775498 PMC7397707

[ref40] McColl-Carboni A.; Dollive S.; Laughlin S.; Lushi R.; MacArthur M.; Zhou S.; Gagnon J.; Smith C. A.; Burnham B.; Horton R.; Lata D.; Uga B.; Natu K.; Michel E.; Slater C.; DaSilva E.; Bruccoleri R.; Kelly T.; McGivney J. B. Analytical Characterization of Full, Intermediate, and Empty AAV Capsids. Gene Ther. 2024, 31, 285–294. 10.1038/s41434-024-00444-2. 38374348 PMC11090809

[ref41] Tam Tran N.; Wl Tai P. Profiling AAV Vector Heterogeneity & Contaminants Using Next-Generation Sequencing Methods. Cell Gene Ther. Insights 2024, 09 (11), 1565–1583. 10.18609/cgti.2023.206.

[ref42] Rayaprolu V.; Kruse S.; Kant R.; Venkatakrishnan B.; Movahed N.; Brooke D.; Lins B.; Bennett A.; Potter T.; McKenna R.; Agbandje-McKenna M.; Bothner B. Comparative Analysis of Adeno-Associated Virus Capsid Stability and Dynamics. J. Virol. 2013, 87 (24), 13150–13160. 10.1128/JVI.01415-13. 24067976 PMC3838259

[ref43] Miller L. M.; Draper B. E.; Barnes L. F.; Ofoegbu P. C.; Jarrold M. F. Analysis of Megadalton-Sized DNA by Charge Detection Mass Spectrometry: Entropic Trapping and Shearing in Nanoelectrospray. Anal. Chem. 2023, 95 (23), 8965–8973. 10.1021/acs.analchem.3c01027. 37267126

[ref44] Wörner T. P.; Aizikov K.; Snijder J.; Fort K. L.; Makarov A. A.; Heck A. J. R. Frequency Chasing of Individual Megadalton Ions in an Orbitrap Analyser Improves Precision of Analysis in Single-Molecule Mass Spectrometry. Nat. Chem. 2022, 14 (5), 515–522. 10.1038/s41557-022-00897-1. 35273389 PMC9068510

[ref45] Wörner T. P.; Bennett A.; Habka S.; Snijder J.; Friese O.; Powers T.; Agbandje-McKenna M.; Heck A. J. R. Adeno-Associated Virus Capsid Assembly Is Divergent and Stochastic. Nat. Commun. 2021, 12 (1), 1642 10.1038/s41467-021-21935-5. 33712599 PMC7955066

