# Supplementary material for: Multimass Analysis of Adeno-Associated Virus Vectors by Orbitrap-Based Charge Detection Mass Spectrometry
Source: Anal Chem. 2024 Oct 10;96(42):17037–46. doi: 10.1021/acs.analchem.4c05229 (PMC11503520; doi:10.1021/acs.analchem.4c05229)
Supplement: Supplementary file 1 — ac4c05229_si_001.pdf [file ac4c05229_si_001.pdf]

Supporting Information

Multi-Mass Analysis of Adeno-Associated Virus Vectors by Orbitrap-Based Charge Detection Mass Spectrometry

Ryoji Nakatsuka[1, 2, 3], Yuki Yamaguchi[1], Kiichi Hirohata[1], Saki Shimojo[1], Makoto Murakami[1], Mark Allen Vergara Rocafort[1], Yasuo Tsunaka[1], Mitsuko Fukuhara[1, 4], Tetsuo Torisu[1], Susumu Uchiyama[1]

[1]Department of Biotechnology, Graduate School of Engineering, Osaka University, 2-1 Yamadaoka, Suita, Osaka 565-0871, Japan

[2]Technology Research Laboratory, Shimadzu Corporation, 1, Nishinokyo-Kuwabaracho Nakagyo-ku, Kyoto 604-8511, Japan

[3]Osaka University Shimadzu Analytical Innovation Research Laboratories, Osaka University, 2-1 Yamadaoka, Suita, Osaka 565-0871, Japan

[4]U-Medico Inc, 2-1 Yamadaoka, Suita, Osaka 565-0871, Japan

* Corresponding author: Susumu Uchiyama, Ph.D. E-mail: suchi@bio.eng.osaka-u.ac.jp

Abstract for Supporting Information.

We have added Figures mentioned in the main text as follow. The order of the contents in this information correspond to the order described in the main text.

CE-SDS analysis of AAV8-VP3-only-EP

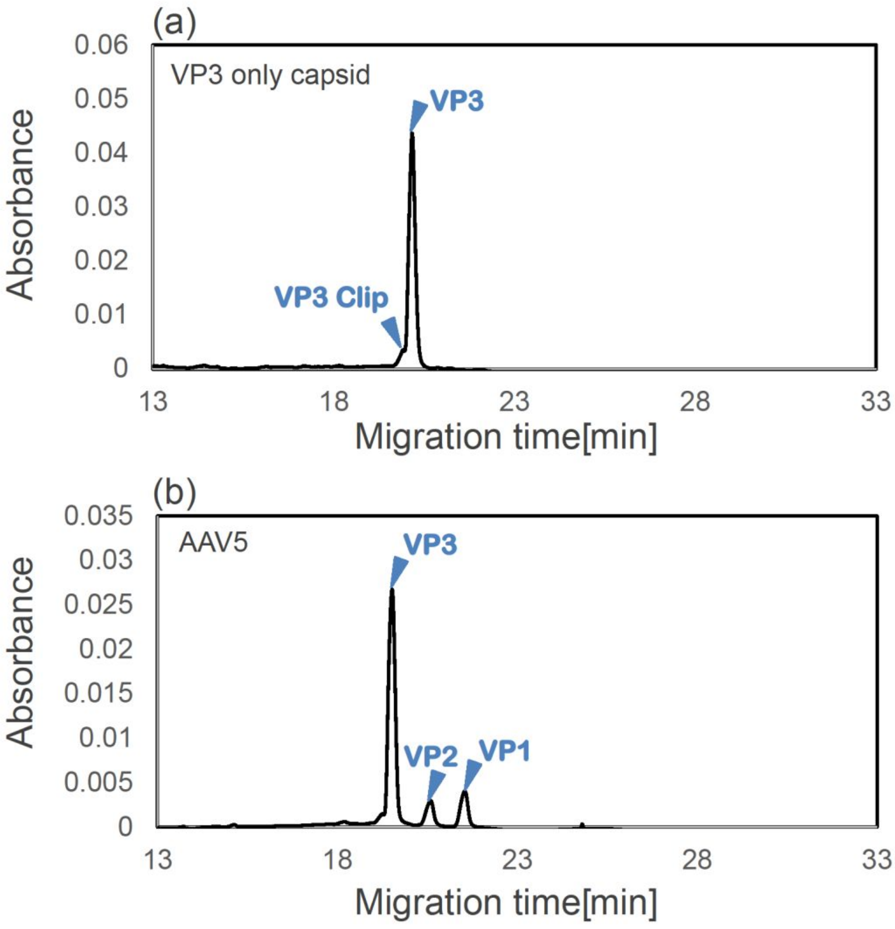

Figure S1. CE-SDS evaluation of AAV8-VP3-only-EP. (a) The VP ratio of the AAV8-VP3-only-EP, manufactured in-house, was measured by CE-SDS. (b) By comparing it with the control sample shown, the absence of VP1 and VP2, which constitute the capsid, was confirmed.

Charge calibration for Orbitrap-CDMS analysis

Charge calibration was performed to comprehend the correlation between the slope of the STORI, which was a Fourier series-like accumulation value of image charge, and the ion charge. In this study, it was necessary to use calibrants with a charge of around +150–170, similar to that of the AAV vector, and with minimal heterogeneity. Therefore, for the charge calibration, GroEL and AAV8-VP3-only-EP were employed. Figure S2 presents the results of the native MS analysis of GroEL 14-mer (801 kDa) and AAV8-VP3-only-EP (3.59 MDa), and the charge calibration. As shown in Figure S2(a) and (b), GroEL and AAV8-VP3-only-EP were completely separated by charge state, showing dominant peaks at +64 to +70 and +148 to +168, respectively. These native spectra were acquired under slightly different conditions compared to CDMS. For GroEL and AAV8-VP3-only-EP, the IST voltage was set to -70 and -80, respectively, and the trapping gas pressure was set to 2 and 1, respectively. As a result of calculating the calibration line using these samples, the calibration coefficient was determined to be 46694.28, as shown in Figure S2(c).

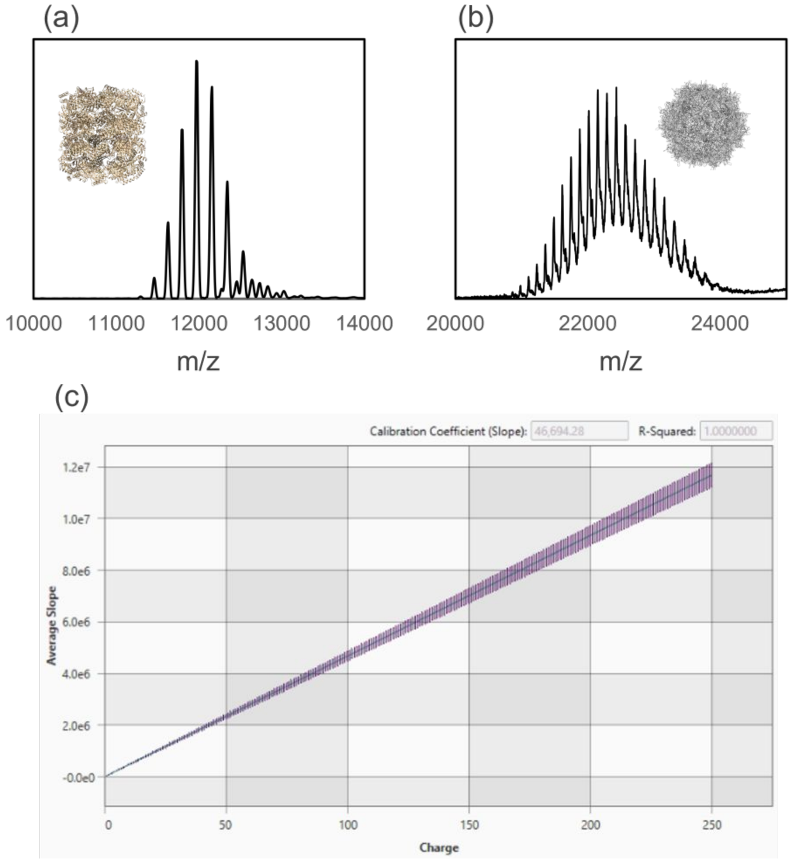

Figure S2. Charge calibration with GroEL and AAV8-VP3-only-EP. Mass-to-charge spectrum in native states of (a) GroEL 14-mer and (b) AAV8-VP3-only-EP. Data were acquired at resolutions of 100k and 50k, respectively. (c) Calibration curve established using CDMS results of GroEL and AAV8-VP3-only-EP.

SV-AUC assessment for EP 100% and FP 100% samples.

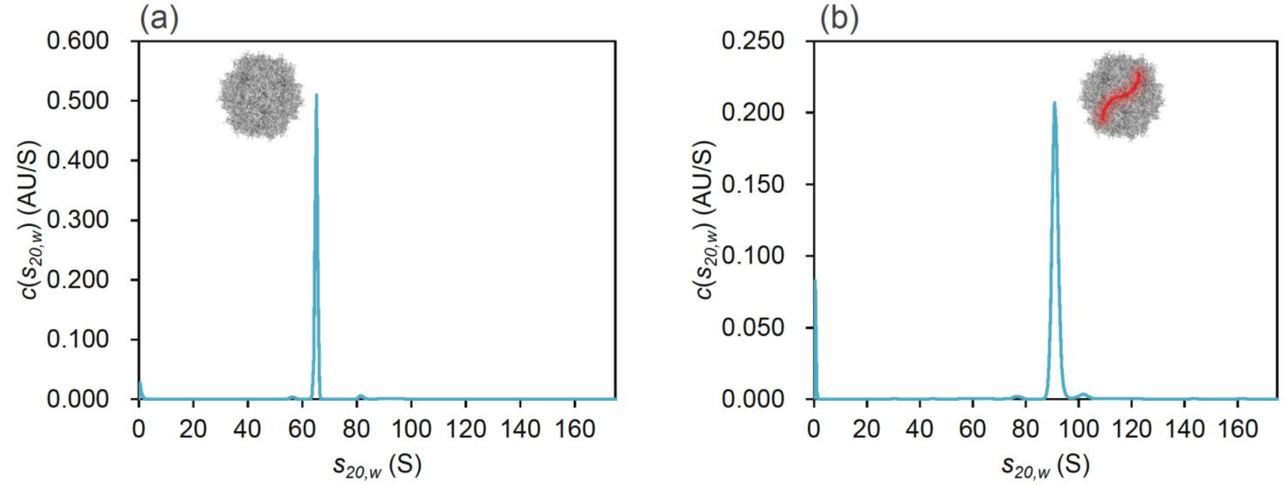

Figure S3. Assessment of EP 100% and FP 100% samples by SV-AUC. EF ratio assessment by SV-AUC for (a) EP 100% and (b) FP 100% sample which also used for assessment of EF ratio quantification were performed. All minor peaks in both results were below 0.038, LOQ of absorbance at UV230 nm in SV-AUC, indicating these samples contains no impurities respectively.

Dependency of AAV capsid disassembly on in-source trapping voltage.

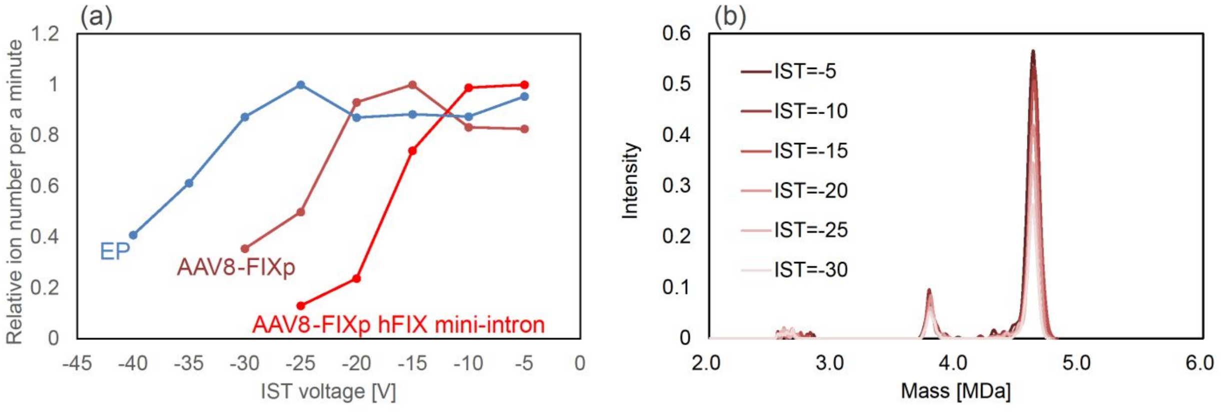

Figure S4. Dependency of AAV capsid disassembly on in-source trapping voltage. (a) The capsid disassembly of EP capsid occurs at larger negative IST voltage and more gradually compared to FP capsid. Additionally, the longer the genome length encapsidated in AAV vectors, the more abrupt the capsid disassembly occurs. (b) The capsid disassembly spectra of AAV8-FIX showed that the FP signal lost due to IST mainly fragments and does not convert significantly into EP.

EP disassemble by dissociation CDMS.

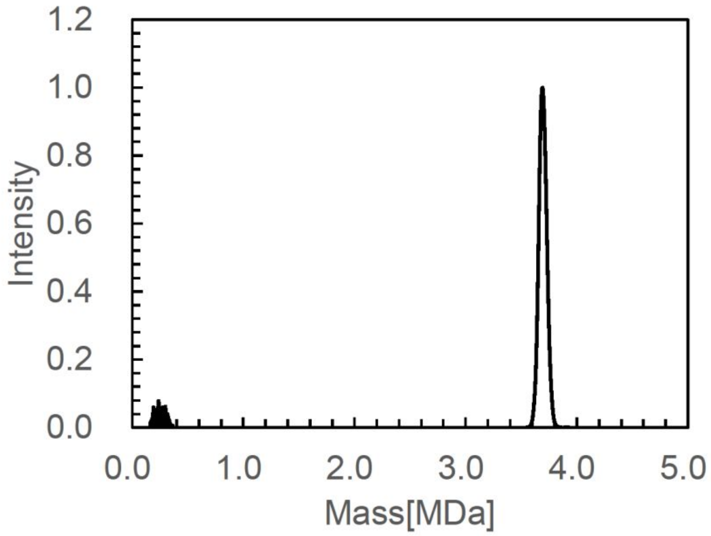

Figure S5. Mass spectrum of disassembled EP capsid by -150 V of IST. When 100% empty capsids are disassembled, there were no signal in the 0.1-1 MDa region, except for AAV fragments.

CDMS analysis of EP 3% sample.

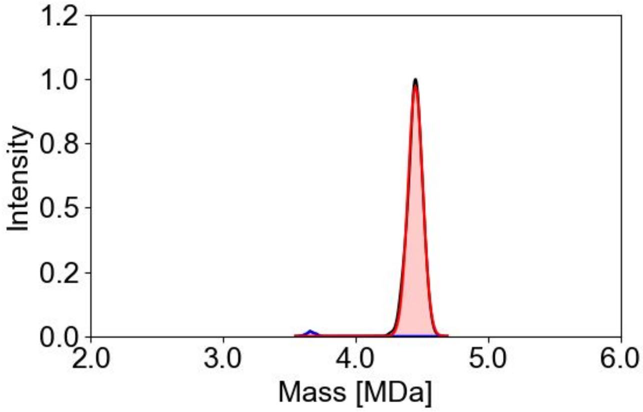

Figure S6. CDMS analysis results of the EP 3% sample. A result of 0.85±0.15% was obtained with N=3. Although it is not a region where relative quantification is possible, it is possible to detect even at EP 3%.

Noise components during MP analysis

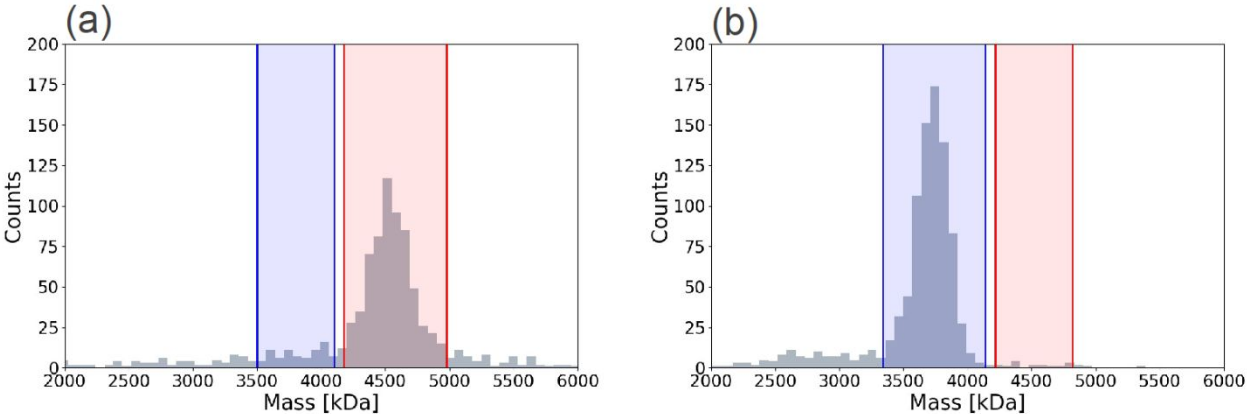

Figure S7. Histograms of EP: FP=0: 100 and 100: 0 analyzed by MP. Noise components appear on the left side of the dominant peak. (a) When FP is 100%, noise occurs in the region where EP exists. (b) Similarly, when EP is 100%, noise also occurs, but it does not affect the count of either EP or FP.

CE-LIF analysis for encapsidated genome length of AAV8-H4C1, AAV8-FIXp, and AAV8-FIXp hFIX mini-intron

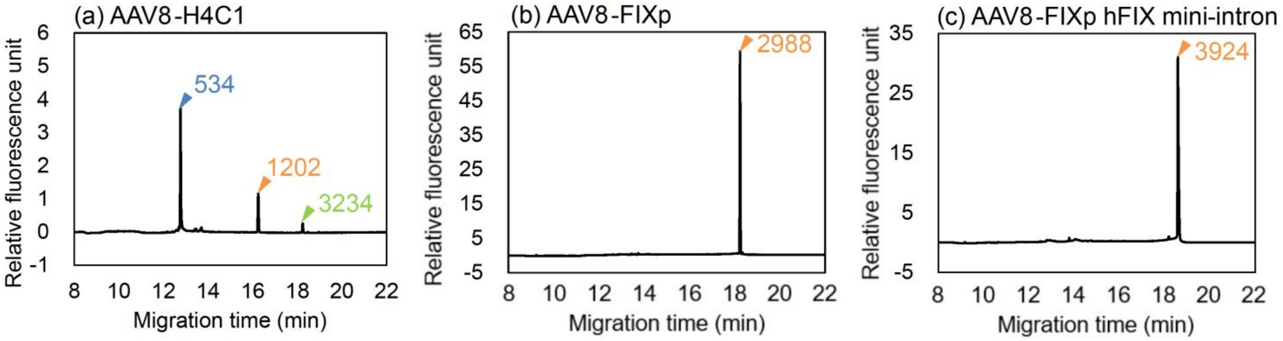

Figure S8. **Evaluation of encapsidated GOI using CE-LIF.** The length of the GOI encapsidated in the AAV vectors was analyzed using CE-LIF. The lengths of AAV8-H4C1, AAV8-FIXp, and AAV8-FIXp hFIX mini-intron were calculated to be (a) 1202, (b) 2988, and (c) 3924 bases, respectively. In addition, from the results in (a), it was found that AAV8-H4C1 encapsidated a DNA sequence of 534 bases (blue arrowhead) and 3234 bases (light green arrowhead), which is shorter and longer, respectively than the GOI at 1202 bases.

Evaluation of encapsidated genome in AAV5-CMV-EGFP

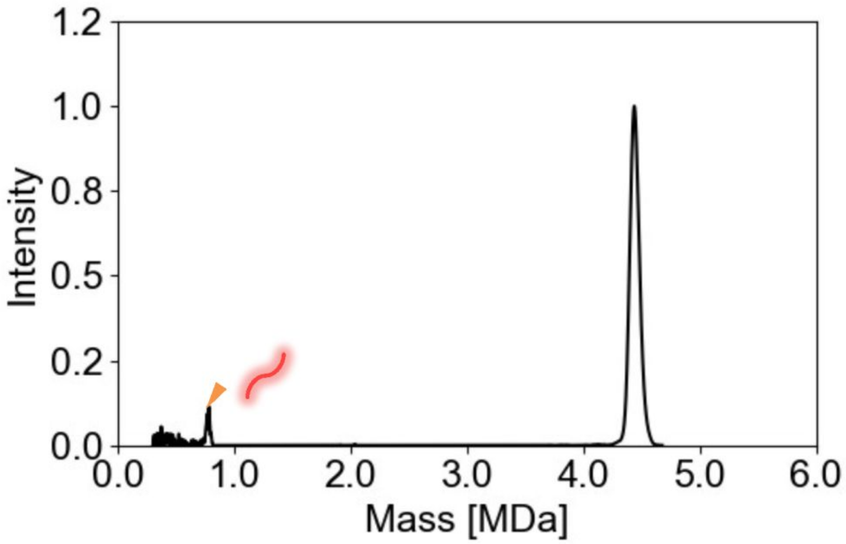

Figure S9. Evaluation of encapsidated genome in AAV5-CMV-EGFP by orbitrap-based CDMS.

VP ratio evaluation by CE-SDS.

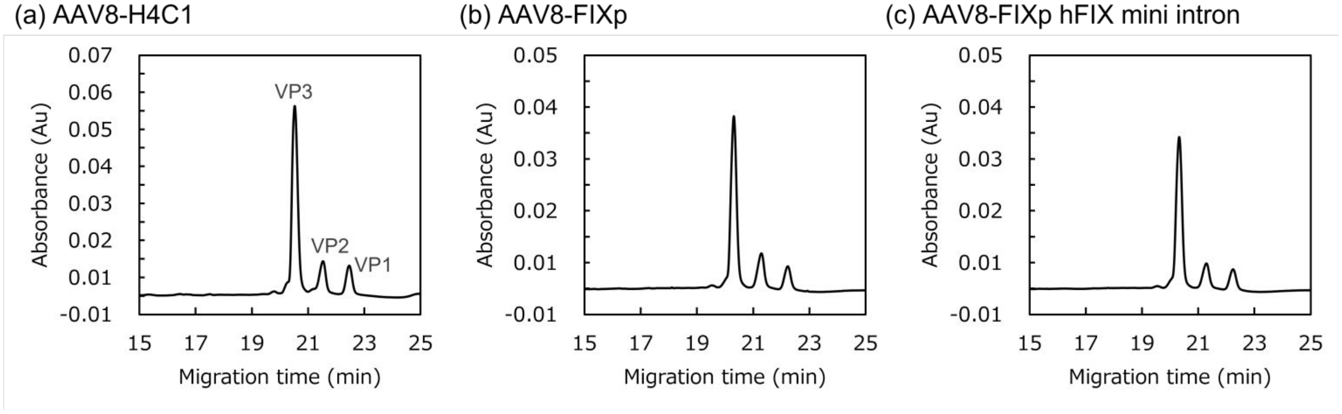

Figure S10. Assessment of VP1, VP2 and VP3 component by CE-SDS. The ratio was calculated by computing the area ratio. For (a) AAV8-H4C1, (b) AAV8-FIXp, and (c) AAV8-FIXp hFIX mini-intron, the VP ratios measured by CE-SDS were 5.1: 7.8: 47.0, 4.6: 9.3: 46.1, and 4.7: 7.5: 47.8, respectively.

Integrity evaluation of AAV8-H4C1, AAV8-FIXp, and AAV8- FIXp hFIX mini-intron by BS-AUC and MP

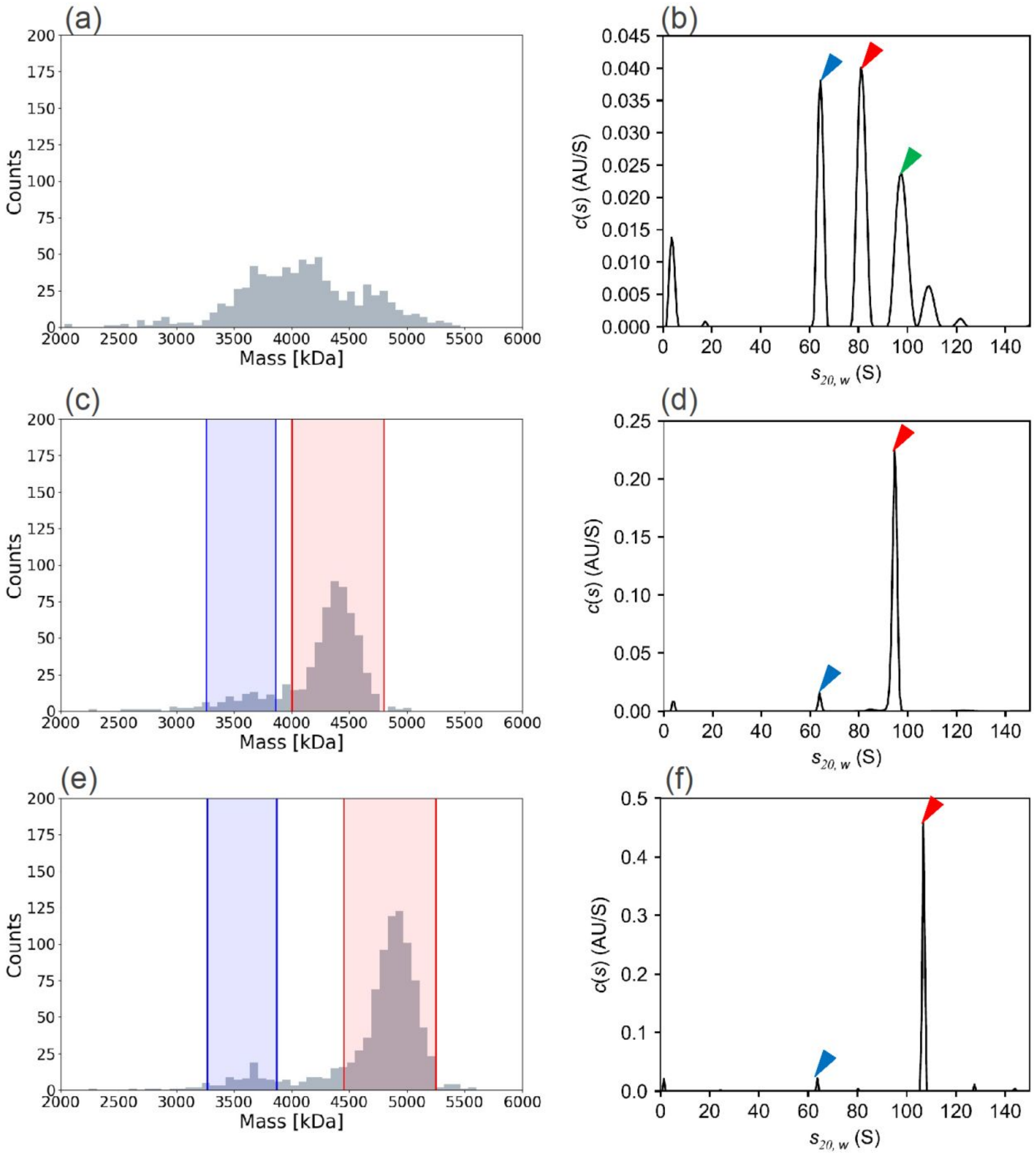

Figure S11. Assessment of E/F ratio by MP and BS-AUC. E/F ratio of (a), (b) AAV8-H4C1, (c), (d) AAV8-FIXp, and (e), (f) AAV8-FIXp hFIX mini-intron was performed analyzed by (a), (c), (e) MP and (b), (d), (f) BS-AUC, respectively. (a), (c), (e) The region enclosed by the blue line represents EP, while the region enclosed by the red line represents FP. (b), (d), (f) The blue, red, and green arrowheads indicate the peaks of EP, FP, and OP, respectively.

The algorithm for estimating VP stoichiometry using mass distribution.

In analytical instruments that measure the mass distribution of samples, such as mass spectrometry, it is believed that this mass distribution reflects the heterogeneity of viral samples. In other words, mass values that can be expressed in integer ratios for VP are assigned to the mass values obtained from the mass spectrum. By calculating a weighted average for each mass value, the stoichiometry of VP can be estimated. The specific algorithm is as follows:

1. Subtract the mass of DNA from the overall mass distribution of FP.
2. Perform Gaussian fitting on the peaks derived from FP (at this point, the mass is approximately that of EP).
3. Assign mass values that can be expressed in integer ratios for VP.
4. Read the intensity values for each assigned mass value.
5. Calculate the averaged stoichiometry by summing the products of the ratio of VP and the intensity values associated with that ratio, and then dividing by the number of accumulations.

$$VPr_x = \frac{1}{n}\sum_{i=1}^{n} VPr_{x,\ i} \cdot G(m_i)$$

(VPr_x: VP ratio, m_i: i-th assigned mass value, $G(m_i)$: Fitting function to represent the mass distribution)
